# Optimization of cellulase production by *Enhydrobacter* sp. ACCA2 and its application in biomass saccharification

**DOI:** 10.3389/fmicb.2015.01046

**Published:** 2015-10-08

**Authors:** Nagaiah Premalatha, Nellaiappan O. Gopal, Polpass Arul Jose, Rangasamy Anandham, Soon-Wo Kwon

**Affiliations:** ^1^Department of Agricultural Microbiology, Agricultural College and Research Institute, Tamil Nadu Agricultural UniversityMadurai, India; ^2^Korean Agricultural Culture Collection, National Academy of Agricultural Science, Rural Development AdministrationJeonju, South Korea

**Keywords:** carboxy methyl cellulose, cellulase, response surface methodology, central composite design, saccharification

## Abstract

Cellulase finds use in saccharification of lignocellulosic agroresidues to fermentable sugars which can be used for production of commercially important metabolites. This study reports endoglucanase (CMCase) production by *Enhydrobacter* sp. ACCA2. The CMCase activity of the strain ACCA2 was successively improved by optimization of range of physical and nutritional parameter in a set of non-statistical and statistical experiments. Initial non-statistical selection of carbon source, incubation time, temperature and pH resulted in 1.07 fold increase of CMCase activity. In a subsequent statistical method, response surface methodology, optimization of medium components such as carboxymethylcellulose, peptone, NaCl, MgSO_4_, K_2_HPO_4_, and (NH_4_)_2_SO_4_ yielded further increase up to 2.39 fold CMCase activity. The cellulolytic potential was evaluated in biomass saccharification with different plant materials and the results revealed that the enzyme produced by strain may have significant commercial values for industrial saccharification process. Moreover, this is the first report of cellulase production by an *Enhydrobacter* spp.

## Introduction

Cellulose biomass is the most abundant and renewable resource and attractive raw material for many industries in production of animal feed (Beauchemin et al., 1995; Lewis et al., 1996; Twomey et al., 2003), manure, paper (Kirk et al., 2002), and fuel (Fujita et al., 2002; Chang, 2007). However, the microcrystalline structure of cellulose challenges the effective utilization of cellulosic biomasses (Lai et al., 2011; Yang et al., 2014). Traditional acid, alkali and heat treatment methods offer good results, but they also render secondary pollutions (Agbor et al., 2011). Use of microbial enzymes has alternatively been proposed as a highly efficient approach for biodegradation of cellulosic biomasses (Fang et al., 2012; Li et al., 2012).

Cellulases, a group of glycosyl hydrolases, including endoglucanase, exoglucanase and β-glucosidase, show distinct enzymatic actions on the breakdown of cellulose (Yang et al., 2014). These cellulose hydrolytic enzymes are produced naturally by a wide range of microbial communities, including bacterial and fungal species of diverse environmental origin (Santhi et al., 2014a). The cellulases derived from different microbial sources show significant difference in their stability, catalytic potential and thereby rate of cellulose breakdown (Liu et al., 2012; Santhi et al., 2014b; Sindhu et al., 2014). Therefore, worldwide research has been focused on isolation and exploitation of new microbial resources for the extraction of cellulolytic enzymes with desirable catalytic potential (Harshvardhan et al., 2013).

*Enhydrobacter*, a rare genus with its single species *Enhydrobacter aerosaccus* LMG 21877^T^ had been proposed by Staley et al. (1987). Later, the genus was discussed to be recognized as a member of the family *Rhodospirillaceae* within the class *Alphaproteobacteria* (Kawamura et al., 2012). Further studies are limited and there is no previous report of cellulolytic potential in *Enhydrobacter*. The newly isolated *Enhydrobacter* sp. ACCA2 is a new member to this rare genus as well as to the cellulolytic bacterial community. Therefore, it is worthy to formulate optimized culture media and conditions, and evaluate its applicability to saccharification of plant biomasses.

Cellulase production is inducible in bacteria, significantly influenced by nutritional composition and physical process parameters such as incubation period, temperature, pH and agitation speed (Maki et al., 2011; Goyal et al., 2014; Yang et al., 2014). Optimization of both nutritional and process parameters can considerably improve cellulase production in bacteria, and plays significant role in development of industrial bioprocess for enzyme production (Sadhu et al., 2014; Singh et al., 2014). An ideal practice to optimize such parameters is RSM. This method employs a statistical experimental design and provides statistically validated predictions to ease the optimization process (Zambare, 2011; Singh et al., 2014).

In the current study, a cellulolytic rare bacterium was isolated and characterized from leaf litter compost collected from a Western Ghats region of India. Culture media and process parameters were optimized using a set of conventional and statistical methods to ensure improved production of cellulases in batch cultures. In addition, the strain was evaluated for its biomass saccharification potential. This is the first report of its kind in rare *Enhydrobacter* species.

## Materials and Methods

### Origin of Cellulolytic Strain ACCA2

Leaf-litter compost samples were collected from Western Ghats region (09° 39′50.7″ N, 077° 18′16.2″ E) of Madurai, India. One gram of the sample was suspended in 99 ml sterile water and used for isolation of cellulase producing bacteria by the dilution pour plate technique in carboxymethyl cellulose agar medium (10 g carboxymethyl cellulose, 1 g K_2_HPO_4_, 0.5 g MgSO_4_, 0.5 g NaCl, 10 g peptone, 0.25 g (NH_4_)_2_SO_4_, pH 6.8–7.0) and incubation at 28 ± 2°C for 3 days. Appeared colonies were screened for cellulolytic activity on cellulose congo*-*red agar media (KH_2_PO_4_ 0.5 g, MgSO_4_ 0.25 g, cellulose 2 g, agar 15 g, Congo-Red 0.2 g, and agar 2 g; distilled water 1 L and at pH 6.8–7.2; Hendricks et al., 1995). Among the pool of cellulolytic isolates, strain ACCA2 showed the highest cellulolytic activity and was selected for further study on its application in cellulose degradation.

### Molecular Identification of Cellulolytic Strain ACCA2

The strain ACCA2 was cultivated in nutrient medium and DNA was extracted according to Sambrook et al. (1989). The gene encoding bacterial 16S rRNA was amplified through PCR with forward primer, 27f: 5′-AGAGTTTGATCCTGGCTCAG-3′ and reverse primer, 1492r: 5′-GGTTACCTTGTTACGACTT-3′ (Lane, 1991). The resulted PCR amplicon was sequenced by fluorescent dye terminators method (ABI Prism ^TM^ Bigdye^TM^ Terminator cycle sequencing ready reaction kit v.3.1) and the products were purified by Millipore-montage dye removal kit. Finally the products were run in an ABI3730XL capillary DNA sequence (50 cm capillary). Nearly complete 16S rRNA gene sequence data obtained from automatic sequencer was aligned and bacterial identity was deduced by using the EzTaxon-e server^1^ (Kim et al., 2012) to ascertain their closest relatives. The 16S rRNA gene sequence obtained from this study was submitted to NCBI with accession number, JX042472.

### Enzyme Assays

Cellulase producing capability of strain ACCA2 was assayed using carboxymethyl cellulose (Sigma, USA) as substrate according to Wood and Bhat (1988). The strain ACCA2 was cultured in CMC broth at 28 ± 2°C for 24 h and the culture supernatant was collected by centrifugation at 12,000 rpm for 10 min. A 200 μL aliquot of supernatant was mixed with 200 μL of 1% (w/v) CMC solution (substrate) and incubated at 50°C for 30 min. Then, 3 ml of dinitrosalicylate (DNS) reagent containing 1% NaOH, 20% Rochelle salt, 2% phenol, 0.005% sodium sulfite and 1% 3,5- dinitrosalicylic acid was added to the mixture and incubated at 100°C for 10 min. The mixture was cooled to room temperature and the absorption was measured at 575 nm against reagent blank. For the development of standard curve, above procedure was followed with known concentration of glucose. One unit (U) of enzyme activity was defined as the amount of enzyme that liberated 1 μmol of reducing sugars per minute under the above conditions (Wood and Bhat, 1988).

### Cellulolytic Activity under Different Carbon Sources

In order to determine the effect of carbon sources on cellulolytic activity of strain ACCA2, different carbon sources such as cellobiose, cellulose, carboxy methyl cellulose, arabinose, galactose and xylose were evaluated individually instead of the carbon source in the nutrient medium (10 g carbon source, 1 g K_2_HPO_4_, 0.5 g MgSO_4_, 0.5 g NaCl, 10 g peptone, 0.25 g (NH_4_)_2_SO_4_). Inoculated culture flasks were incubated at 28 ± 2°C in a rotary shaker for 24 h, the culture supernatants were collected and assayed for culluloytic activity as described above.

### Cellulolytic Activity under Different Physiological Conditions

Cellulase activity (assayed using CMC) was determined in different incubation times, temperatures and initial pH. The strain ACCA2 was inoculated into CMC broth and then incubated at 28 ± 2°C for different time period (1, 2, 3, 4, and 5 days). After the respective incubation period, the culture was centrifuged and 200 μl of culture supernatant was assayed for CMCase activity. Similar to this, the strain ACCA2 was cultured at different temperature (25, 30, 35, 45, 55°C) for 2 days and in different initial pH (4.5, 5.5, 6.5, 7.5, and 8.5), and CMCase activity was determined to identify the optimum temperature and pH.

### Statistical Optimization of Media Components for CMCase Production

To determine the optimum levels of growth medium components for CMCase production, RSM was employed with a CCD. The range and the levels of media components investigated in this study are given in **Table 1**. A total of 52 experiments were conducted with five coded levels (-1.682, -1, 0, +1, and +1.682) as given in **Table 2**. The CMCase activity (response) was assayed as described in earlier section. The response value (Y) in each trial was the average of the duplicates. The Design expert trial version 8.0.1 was used for development of design matrix and statistical analysis of the data. The regression analysis is performed to estimate the response function as a second order polynomial (Wang et al., 2011):

**Table 1:** Factors involved according to CCD in RSM for optimization of cellulase production.

Factors	Coded and actual values				
	-1.682	-1	0	+1	+1.682
CMC	2.174	5	10	15	17.825
Peptone	2.174	5	10	15	17.825
NaCl	0.130	0.3	0.6	0.9	1.069
MgSO_4_	0.130	0.3	0.6	0.9	1.069
(NH_4_)_2_SO_4_	0.065	0.15	0.3	0.45	0.534
K_2_HPO_4_	0.217	0.5	1	1.5	1.782

**Table 2:** Central composite design matrix, observed, and predicted responses.

Run	Media components, codes, and coded values						Cellulase activity (U/mL)	
	CMC (A)	Peptone (B)	NaCl (C)	MgSO_4_ (D)	(NH_4_)_2_SO_4_ (E)	K_2_HPO_4_ (F)	Observed	Predicted
1	-1	-1	-1	-1	-1	-1	2.30	2.29
2	+1	-1	-1	-1	-1	+1	2.70	2.67
3	-1	+1	-1	-1	-1	+1	2.00	2.16
4	+1	+1	-1	-1	-1	-1	2.70	2.84
5	-1	-1	+1	-1	-1	+1	2.00	2.01
6	+1	-1	+1	-1	-1	-1	2.30	2.34
7	-1	+1	+1	-1	-1	-1	2.90	2.78
8	+1	+1	+1	-1	-1	+1	2.59	2.75
9	-1	-1	-1	+1	-1	+1	3.00	3.20
10	+1	-1	-1	+1	-1	-1	2.10	2.09
11	-1	+1	-1	+1	-1	-1	2.30	2.18
12	+1	+1	-1	+1	-1	+1	2.50	2.55
13	-1	-1	+1	+1	-1	-1	3.95	4.15
14	+1	-1	+1	+1	-1	+1	2.30	2.20
15	-1	+1	+1	+1	-1	+1	2.20	2.24
16	+1	+1	+1	+1	-1	-1	2.50	2.58
17	-1	-1	-1	-1	+1	+1	2.20	2.19
18	+1	-1	-1	-1	+1	-1	2.30	2.32
19	-1	+1	-1	-1	+1	-1	2.00	2.16
20	+1	+1	-1	-1	+1	+1	2.30	5.16
21	-1	-1	+1	-1	+1	-1	2.10	2.11
22	+1	-1	+1	-1	+1	+1	2.60	2.79
23	-1	+1	+1	-1	+1	+1	2.50	2.57
24	+1	+1	+1	-1	+1	-1	4.30	4.16
25	-1	-1	-1	+1	+1	-1	1.80	1.70
26	+1	-1	-1	+1	+1	+1	2.60	2.78
27	-1	+1	-1	+1	+1	+1	2.20	2.22
28	+1	+1	-1	+1	+1	-1	2.30	2.35
29	-1	-1	+1	+1	+1	+1	2.40	2.32
30	+1	-1	+1	+1	+1	-1	2.20	2.11
31	-1	+1	+1	+1	+1	-1	2.40	2.49
32	+1	+1	+1	+1	+1	+1	3.10	3.17
33	-1.682	0	0	0	0	0	1.94	1.72
34	+1.682	0	0	0	0	0	2.50	2.31
35	0	-1.682	0	0	0	0	2.20	2.05
36	0	+1.682	0	0	0	0	2.80	2.55
37	0	0	-1.682	0	0	0	2.40	2.15
38	0	0	+1.682	0	0	0	2.50	2.34
39	0	0	0	-1.682	0	0	4.80	4.59
40	0	0	0	+1.682	0	0	4.50	4.30
41	0	0	0	0	-1.682	0	3.30	2.97
42	0	0	0	0	+1.682	0	3.20	3.12
43	0	0	0	0	0	-1.682	3.50	3.48
44	0	0	0	0	0	+1.682	4.10	3.71
45	0	0	0	0	0	0	2.70	3.71
46	0	0	0	0	0	0	2.50	3.71
47	0	0	0	0	0	0	3.70	3.71
48	0	0	0	0	0	0	3.10	3.71
49	0	0	0	0	0	0	2.80	3.71
50	0	0	0	0	0	0	2.20	3.71
51	0	0	0	0	0	0	2.90	3.71
52	0	0	0	0	0	0	3.00	3.71

$$Y\;=\;\beta_{0}+\Sigma \beta_{i}X_{i}+\Sigma \beta_{ij}X_{i}X_{j}+\Sigma \beta_{ii}X_{i}{}^{2}$$

where *Y* is the predicted response, *β_0_* is the intercept term, *β_i_* is the linear coefficient, *β_ij_* is the quadratic coefficient and *β_ii_* is the interaction coefficient.

Analysis of variance (ANOVA) was adopted to determine statistical adequacy of the model. Overall model significance was validated using Fisher’s *F*-test and its associated probability. The quality of the polynomial model equation was judged statistically through coefficient of determination (*R^2^*) and adjusted *R^2^*. Three-dimensional response surface plots were drawn to illustrate the relationship between the responses and the experimental levels of each independent variable.

### Experimental Validation

To validate the statistical model and its predictions, experiments have been conducted separately with optimized and un-optimized levels of medium components in shake-flasks. Physical parameters were kept at observed optimal levels. After incubation, culture supernatant was collected by centrifugation at 12,000 rpm for 10 min and assayed for CMCase activity as described before.

### Plant Biomass Materials and Saccharification

Different plant biomasses such as sorghum leaf, sorghum stem, bamboo, cumbu leaf, and cumbu stem were collected and dried using tunnel drier to get brittle texture. The plant biomasses were then chopped into small pieces, powdered and sieved through 0.7–1.0 mm mesh sieves. One gram of the sieved biomass substrate was added to the 100 mL medium containing optimized levels of nitrogen source and mineral salts [1 g K_2_HPO_4_, 0.5 g MgSO_4_, 0.5 g NaCl, 10 g peptone, 0.25 g (NH_4_)_2_SO_4_] and autoclaved at 110°C with 15 lbs pressure for 20 min. The cellulolytic strain, ACCA2 was inoculated and allowed for saccharification reaction at 30°C with orbitary shaking at 120 RPM. Culture supernatants were withdrawn once in every 24 h to monitor the release of reducing sugar pattern. The experiments were performed in triplicates and the means of the replicates were subjected to statistical analysis. Saccharification (%) was calculated as described by Uma et al. (2010).

$$\mathrm{Saccharification}(\%)\;=\;\frac{Reducing sugar formed\times 0.9}{Cellulose content in Biomass}\;\times \;100$$

## Result

### Biochemical and Molecular Identification of Cellulolytic Strain ACCA2

Cellulolytic strain ACCA2 was characterized and identified as *Enhydrobacter* species in series of biochemical and molecular methods. The strain had rough, opaque, and gray or yellowish colonies of approximately 1.0 mm in diameter after 36 h growth at 28°C on cellulose supplemented medium. The cells were Gram-positive, non-motile cocci, have primary mycelium with no spore and exhibited aerobic growth. The strain ACCA2 hydrolyzed the starch, utilized the citrate, reduced the nitrate and produced catalase. Furthermore, it showed negative results in starch utilization and methyl red-Voges-Proskauer tests.

The obtained 16S rRNA gene sequence of the cellulolytic strain ACCA2 was 1477 bp long and was submitted to GenBank under accession number JX042472.1. The 16S rRNA gene based identification using EzTaxon^2^ showed that the degree of sequence similarity of this strain was 99.8% to *E*. *aerosaccus* LMG 21877^T^. The strain ACCA2 has been deposited in the Korean Agricultural Culture Collection (KACC) under the number of KACC 18489.

### Effect of Carbon Sources on CMCase Production

**FIGURE 1:**
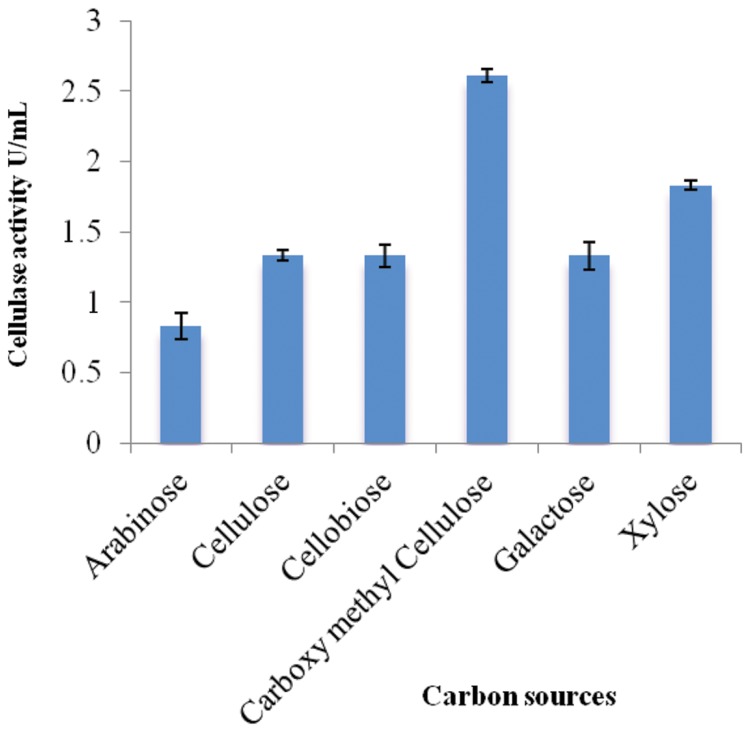
**Effect of carbon sources on cellulase activity of *Enhydrobacter* sp. ACCA2.** The strain ACCA2 was cultured with a range of simple and complex carbon sources as sole carbon source and the cellulase activity was assayed in triplicates. The presented values are the mean and standard deviation.

Different carbon sources were examined to determine their effects on cellulolytic efficiency of strain ACCA2 (**Figure 1**). The results showed that strain ACCA2 could utilize a range of carbon sources, and a maximum cellulose activity (2.61 U/mL) was observed when CMC was used as the sole carbon source. However, moderate CMCase activity (0.83–1.83 U/mL) was observed with cellulosic substances such as cellobiose, cellulose, arabinose, galactose, and xylose. CMC was found to be optimal and used as the carbon source in further experiments to optimize physical conditions such as temperature, pH, and incubation time.

### Optimal Culture Conditions

**FIGURE 2:**
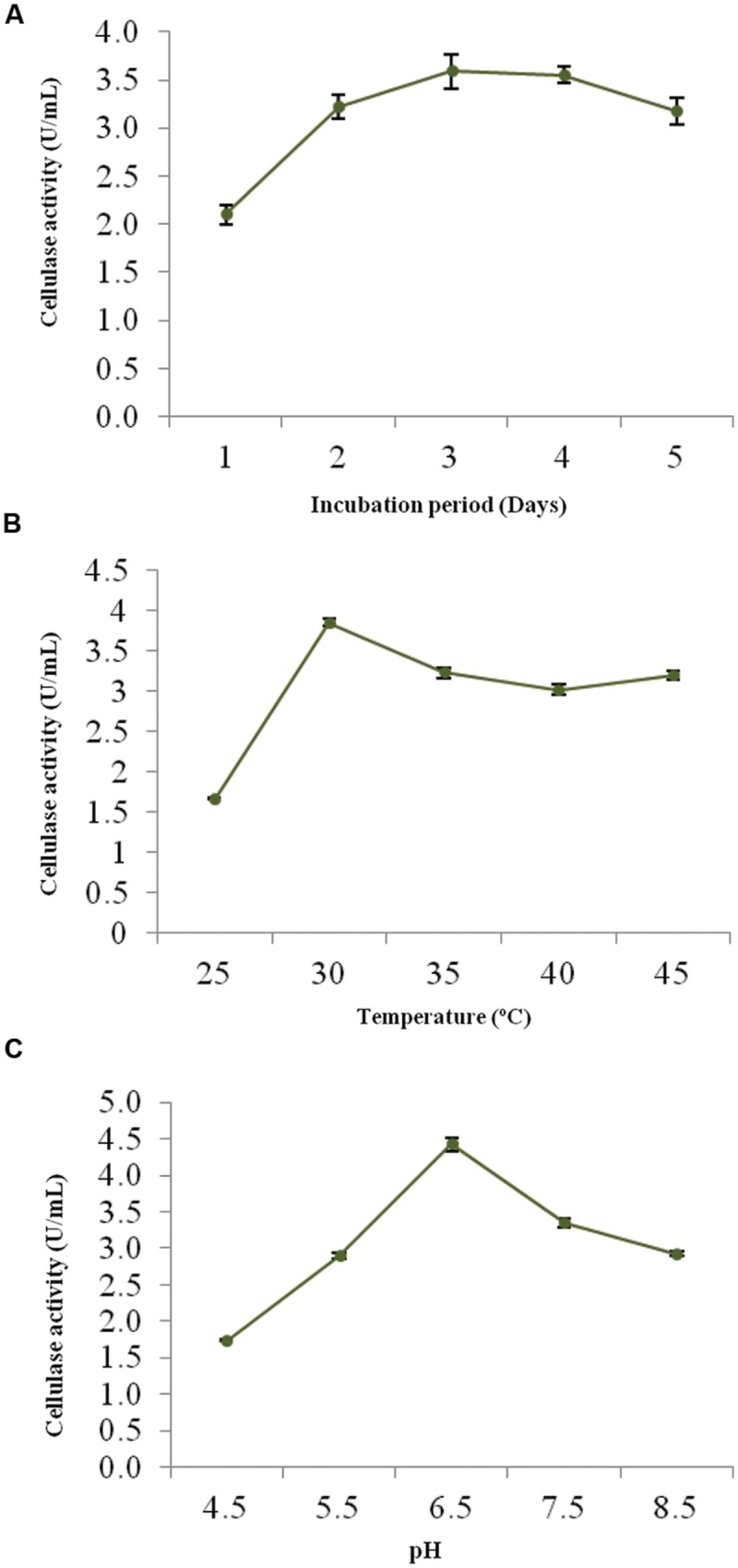
**Effects of process parameters on CMCase activity of *Enhydrobacter* sp. ACCA2.** The strain ACCA2 was cultured under different incubation times **(A)**, temperature **(B)** and initial pH **(C)**, and CMCase activity was assayed in triplicates. The presented values are the mean and standard deviation.

The optimum incubation period, temperature and pH were determined to ensure the better growth and maximum cellulolytic enzyme production. **Figures 2A–C** shows the effect of these physical parameters on CMCase activity of strain ACCA2.

The CMCase activity of strain ACCA2 was measured at various incubation times to determine an optimal one. The results (**Figure 2A**) showed that ACCA2 produced CMCase throughout the incubation period 1–5 days; however, maximum CMCase activity was (3.5 U/mL) with 3 days of incubation. In the further steps of optimization, the incubation period was fixed to 3 days.

To determine the effect and choose an ideal incubation temperature for cellulase production, the culture medium was inoculated and incubated in temperature ranging from 25 to 45°C (**Figure 2B**). The maximum CMCase activity (3.85 U/mL) was recorded at 30°C. Further increase in temperature diminished the activity of ACCA2, however, cellulolytic potential was found to be maintained at moderate level (3.0 U/mL) up to 45°C. In the further steps of optimization, the incubation temperature was kept at 30°C.

The minimum CMCase activity (1.74 U/mL) was observed at an initial medium pH of 4.0, while the maximum (4.42 U/mL) was observed at pH 6.5 (**Figure 2C**). Further increase in initial pH of culture medium caused decrease in CMCase activity. In the further steps of optimization, the initial pH of the medium was kept at 6.5.

### Statistical Optimized Medium for Cellulase Production

The concentration of media components such as CMC, peptone, NaCl, MgSO_4_, (NH_4_)_2_SO_4_ and K_2_HPO_4_ were optimized by RSM with CCD. The statistical combination of the test variables (media components) along with predicted and observed responses (CMCase activity) are presented in **Table 2**. The statistical analysis yielded a regression equation, which shows the empirical relationship of response and test variables. The second-order polynomial equation for cellulolytic activity (*Y*) was as follows:

$$\begin{array}{c} \begin{array}{c} \begin{array}{c} \begin{array}{c} Y\;=\;3.17+0.19A+0.16B+0.060C\;-0.092D+0.049E\\ +0.073F+0.23AB-0.10AC-0.23AD+0.25AE \end{array}\\ \;+0.13AF+0.011BC-0.21BD+0.21BE+7.500E \end{array}\\ -003BF+0.077CD-7.500E-003CE-0.24CF\\ -0.18DE-7.500E-003DF+0.16EF-0.{47A}^{2} \end{array}\\ -0.{36B}^{2}-0.{38C}^{2}+0.{52D}^{2}-0.{050E}^{2}+0.{18F}^{2} \end{array}$$

where *Y* is the cellulolytic activity (U/mL) and A, B, C, D, E, and F are test variables.

The statistical significance of the model equation was inspected by ANOVA and the results are given in **Table 3**. It demonstrated that the model is highly significant and is evident from *F*-value of 11.18 and very low probability *P*-value of <0.0001. The insignificant lack of fit value (1.25) of the model suggested that the obtained experimental data were in good fit. The predicted *R*^2^ of 0.7520 is in reasonable agreement with the adjusted *R*^2^ value of 0.8435. Moreover, *R*^2^ value was found to be 0.9264 and indicated that the model can explain 92.6 % of total variations. In concise, the developed experimental design for predicting cellulolytic activity of ACCA2 was found to be accurate in optimizing the selected medium components.

**Table 3:** Analysis of variance (ANOVA) of quadratic polynomial model and significance test.

Source	Sum of squares	df	Mean square	*F*-value	*p*-value Prob > F	Significance
Model	27.45	27	1.02	11.18	<0.0001	Significant
Residual	2.18	24	0.091			
Lack of fit	1.25	17	0.074	0.56	0.8475	Not significant
Pure error	0.93	7	0.13			
Cor total	29.63	51				

**FIGURE 3:**
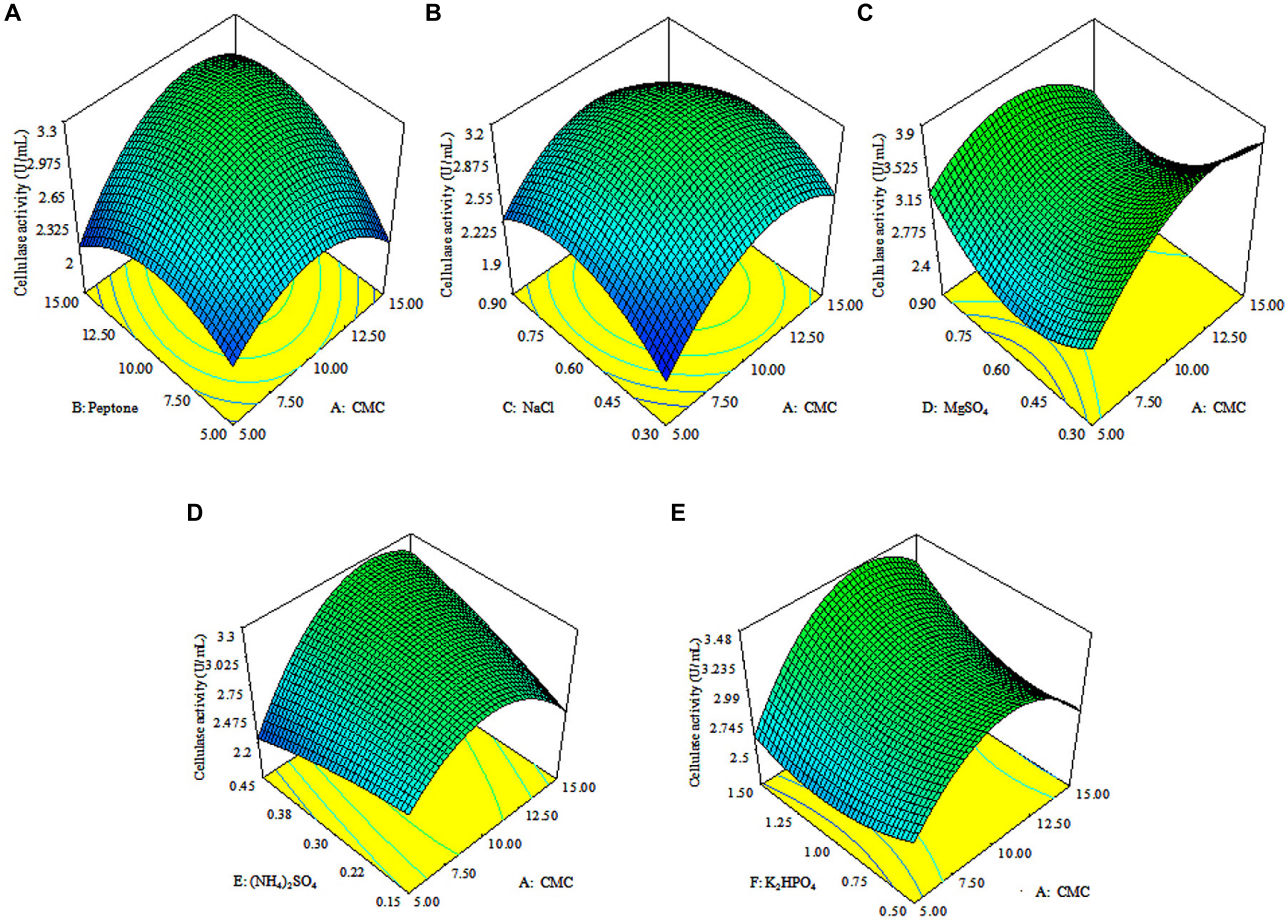
**Response surface plots.** The 3D plots showing individual and interactive effects of variables on CMCase activity of *Enhydrobacter* sp. ACCA2, effect of CMC and peptone **(A)**, effect of CMC and NaCl **(B)**, effect of CMC and MgSO_4_ **(C)**, effect of CMC and (NH_4_)_2_SO_4_ **(D)**, effect of CMC and K_2_HPO_4_ **(E)**.

The 3D response surface plots were drawn to illustrate the individual and interactive effects of media components (**Figure 3**). The 3D plots clearly showed that the maximum cellulolytic activity should occur with moderate concentration of CMC, peptone, (NH_4_)_2_SO_4_ and K_2_HPO_4_, and lowest concentrations of MgSO_4_ and NaCl.

In a numerical optimization, the quadratic model predicted the maximum CMCase activity of 8.20 U/mL, which can be achieved with optimal values of media components: 15 g of CMC, 14.45 g of peptone, 0.44 g of NaCl, 0.31 g of MgSO_4_, 0.45 g of (NH_4_)_2_SO_4_, and 1.5 g of K_2_HPO_4_ in 1 liter of distilled water.

Validation of the final optimized media was done using shake flask experiments with the predicted optimum values of different parameters. Experimental enzyme activity for optimized media was observed to be 8.865 ± 0.043 U/mL, which is an excellent agreement with the predicted value of 8.20 U/mL. It indicated that the developed model was accurate and reliable for predicting the production of CMCase by *Enhydrobacter* sp. ACCA2.

### Overview of Improved Cellulolytic Potential

The overview of obtained enhancement in cellulolytic potential of *Enhydrobacter* sp. ACCA2 is summarized in **Table 4**. After the successive optimization steps, the obtained enhancement of CMCase activity (8.86 U/mL) was about 2.39 fold in comparison to that of 2.61 U/mL obtained under unoptimized conditions and medium components. The increment in activity after optimization of physical parameters alone was 1.07 fold and further optimization of medium components resulted 2.39 fold.

**Table 4:** Successive levels of experiments and achieved enhancement in cellulolytic potential of strain ACCA2.

Stages	Constant parameter	Variable parameter	Range	Suitable/optimum	Maximum activity (U/mL)
Level 1	Basal medium + Incubation time + Temperature + pH	Carbon Source		CMC	2.61
Level 2	Basal medium + CMC + Temperature + pH	Incubation time	1 – 5 days	3 days	3.59
Level 3	Basal medium + CMC + Optimum incubation time + pH	Temperature	25 – 45°C	30°C	3.85
Level 4	Basal medium + CMC + Optimum incubation time + Optimum temperature	pH	4.5 – 8.5	6.5	4.4
Level 5	Optimum incubation time + Optimum temperature + Optimum pH	CMC	2.174 – 17.825 g/L	15.00 g/L	8.86
		Peptone	2.174 – 17.825 g/L	14.45 g/L	
		NaCl	0.130 – 1.069 g/L	0.44 g/L	
		MgSO_4_	0.130 – 1.069 g/L	0.31 g/L	
		(NH_4_)_2_SO_4_	0.150 – 0.534 g/L	0.45 g/L	
		K_2_HPO_4_	0.500 – 1.782 g/L	1.50 g/L	

### Plant Biomass Saccharification

**FIGURE 4:**
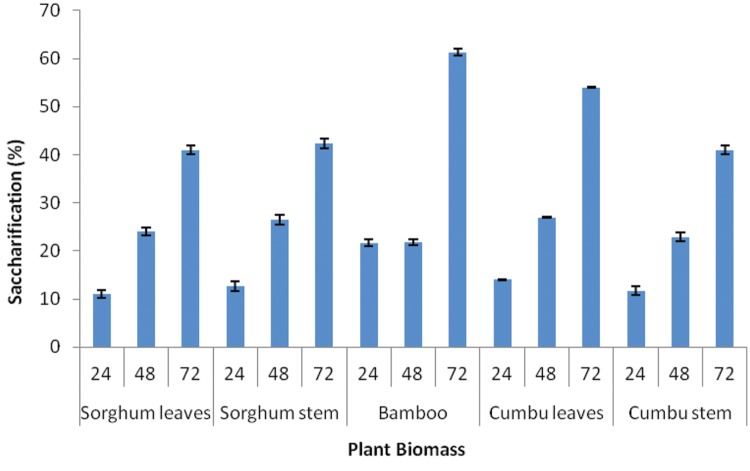
**Biomass saccharification potential of *Enhydrobacter* sp. ACCA2.** The strain ACCA2 was inoculated separately with plant biomasses such as sorghum leaf, sorghum stem, bamboo, cumbu leaf and cumbu stem, and allowed for saccharification reaction. Release of reducing sugar was monitored at fixed time intervals and percentage of saccharification was calculated.

Saccharification of different plant biomasses by *Enhydrobacter* sp. ACCA2 was determined in percentage at three time intervals. **Figure 4** illustrates the percentage of saccharification achieved for different plant biomasses such as sorghum leaf, sorghum stem, bamboo, cumbu leaf, and cumbu stem. The results revealed that strain ACCA2 has potential to saccharify all the tested plant biomasses. Their enzymatic saccharification level varied from 12 to 61.33% for different plant biomasses at different time intervals. Maximum saccharification (61.33 %) was observed for bamboo in third day of incubation.

## Discussion

Exploitation of cellulolytic microorganisms has been recognized as an environmental friendly alternate to chemical processing of cellulose materials for industrial applications. Several cellulolytic microorganisms have been isolated from diverse environments, such as soil (Soares et al., 2012; Sethi et al., 2013), organic waste (Eida et al., 2012; Ghio et al., 2012), gut (Jyotsna et al., 2010; Dantur et al., 2015), animal waste (Singh et al., 2013) marine sediments (Ji et al., 2012; Harshvardhan et al., 2013; Santhi et al., 2014a) and seaweeds (Santhi et al., 2014b). However, the present enzyme toolbox is still inadequate to meet the industrial demands (Chakraborty et al., 2009; Singh et al., 2014), and therefore efforts being continued to isolate potent strains from unexplored environments (Dalmaso et al., 2015). In this study, a rare cellulolytic bacterium was isolated from leaf-litter compost sample. The biochemical characteristics of the strain ACCA2 were found to be comparable to the reported characteristics of *E*. *aerosaccus* LMG 21877^T^ (Staley et al., 1987). Based on its 16S rRNA gene sequence and the biochemical characteristics, strain ACCA2 was designated as *Enhydrobacter* sp. ACCA2. Recent metagenomic studies revealed that members of *Enhydrobacter* occur in diverse environments (Lee et al., 2012; Sharma et al., 2014), however, members of *Enhydrobacter* have not been realized in the culture-dependent methods. This study introduces a cultivable cellulolytic species affiliated to this genus for the first time.

Cellulase is an inducible enzyme (Elisashvili et al., 1999; Zhang et al., 2009). Type of carbon source is an important factor which affects inducible production of cellulase in microorganisms (Yang et al., 2014). In the present study, different carbon sources were screened for their influence on strain ACCA2 and the results revealed that CMC favors maximum cellulase activity when compared to other tested carbon sources. Similar positive effects of CMC have also been reported in other bacteria (Deka et al., 2011; Singh et al., 2014). Other factors which influence the extracellular enzyme production include incubation period, temperature, pH and concentration of individual media components. The fermentation time requirement of ACCA2 for the maximum cellulase activity is comparable to that of different cellulolytic microorganisms (Rastogi et al., 2010; Acharya and Chaudhary, 2011; Sadhu et al., 2013). The production of cellulase at earlier stages of fermentation showed that strain ACCA2 can be useful for large-scale cellulase production. In the case of incubation temperature, maximum cellulase production was observed at 30°C. The initial pH requirements have been reported to vary among bacteria recovered from different sources (Yang et al., 2014). Through this successive selection of carbon source, incubation time, temperature and pH, 1.07 fold increase of cellulase activity was achieved for the strain ACCA2.

Concentration variations of media components exert significant effect on extracellular cellulase production. Therefore, optimization of individual medium components has been considered as a measure to reduce the production cost of cellulase by microorganisms (Sukumaran et al., 2005; Singh et al., 2014). RSM is a well-recognized statistical practice that employs cost effective experimental designs and offers statistical predictions and evaluations (Singh and Kaur, 2012; Jose and Jebakumar, 2014). In the present study, RSM was applied with CCD to optimize media components such as CMC, peptone, NaCl, MgSO_4_, (NH_4_)_2_SO_4_, and K_2_HPO_4_ to improve the cellulase production by *Enhydrobacter* sp. ACCA2. The RSM eased the process of optimization in 54 trials as it involves rapid screening of the optimal levels with lesser consumption of materials and efforts (Singh et al., 2014; Rajeswari et al., 2015).

The developed model was found to be very effective in optimizing the selected medium components evident from *R*^2^ value 0.9264. The closer *R*^2^ is to 1, the stronger is the model to predict the response (Chen et al., 2013). The observed *R*^2^ value was comparable with the earlier reports (Wang et al., 2011; Rajeswari et al., 2015).

A valid optimization of microbial enzyme production is possible with the implication of the 3D plots which allows direct visualization of individual and interactive influence of variables (Wang et al., 2011; Singh et al., 2014). In this study, 3D plots clearly showed that the maximum cellulase activity possibly occur with moderate concentrations of CMC, peptone, (NH_4_)_2_SO_4_ and K_2_HPO_4_, and lowest concentrations of MgSO_4_ and NaCl. The results concur with earlier studies demonstrating the significant roles of organic and inorganic nutrients (Li et al., 2008; Geetha and Gunasekaran, 2010; Deka et al., 2011; Kim et al., 2011; Singh et al., 2014). The effects of media components and cellulase production have not been studied in any of *Enhydrobacter* species. However, the positive induction of cellulase production by moderate concentrations of CMC and peptone has been documented in other bacteria. Deka et al. (2011) have reported a steep increase of cellulase activity in *Bacillus subtilis* AS3 under higher concentration (18 g/L) of CMC. Recently, Singh et al. (2014) revealed that maximum CMCase production has been induced by 19.05 g/L of CMC in *Bacillus amyloliquefaciens* SS35. In the current study, maximum cellulolytic activity was induced in *Enhydrobacter* sp. ACCA2 by 15 g/L of CMC.

In successive optimization steps, the obtained enhancement of cellulase activity was about 2.39 fold in comparison to that of obtained under unoptimized conditions and medium components. In fact, optimization of medium components contributed more to the improved production of cellulase. Thus, the results obviously supported previous conclusions of other researchers (Deka et al., 2013; Singh et al., 2014) that the concentration of major medium components is the principal governing factor for cellulase production.

The ability of bacterial in saccharification of inexpensive cellulosic materials such as wheat straw, corn stover, sugarcane, and rice bran has been reported by several researchers (Lee et al., 2008; Harshvardhan et al., 2013; Santhi et al., 2014b; Dantur et al., 2015). In this study, the cellulolytic strain ACCA2 was evaluated for its saccharification potential to plant biomasses without pretreatment. The results revealed that the strain ACCA2 has significant saccharification potential for all the tested plant biomasses. The strong crystalline structure of cellulose, complex hemicelluloses and lignin contents of the crop residues and tree leaf-litters limits accessibility of plant biomass to hydrolytic enzymes (Santhi et al., 2014b). The degradation effect exerted by ACCA2 on the plant biomasses without pretreatment depicted that the strain ACCA2 has complex enzyme activity with lignocellulolytic effect and saccharification potential.

## Conclusion

A cellulolytic strain previously isolated from leaf litter compost was characterized to molecular level and identified as *Enhydrobacter* sp. ACCA2. In the successive optimization experiments, both physical and medium components were optimized and 2.39 fold increase of cellulytic activity was obtained. CMC, peptone, (NH_4_)_2_SO_4_ and K_2_HPO_4_ were found to be the significant inducing factors for enzyme production. These results indicated that the model developed for maximizing cellulase production by *Enhydrobacter* sp. ACCA2 is reliable and accurate. Subsequently, the bacterial cellulase employed for saccharification of different plant biomass materials and the results suggested that the strain can be further studied and considered for biomass saccharification and allied industrial applications. The present study, thus, introduces cellulase producing member to the genus *Enhydrobacter* and clearly demonstrated optimization strategy for enhanced production of cellulase for biomass saccharification.

## Conflict of Interest Statement

The authors declare that the research was conducted in the absence of any commercial or financial relationships that could be construed as a potential conflict of interest.

## Footnotes

1 http://eztaxon-e.ezbiocloud.net/

2 http://www.ezbiocloud.net

## Abbreviations

CCD: central composite design
CMC: carboxy methyl cellulose
RSM: response surface methodology
